# On the Embodiment of Negation in Italian Sign Language: An Approach Based on Multiple Representation Theories

**DOI:** 10.3389/fpsyg.2022.811795

**Published:** 2022-08-30

**Authors:** Valentina Cuccio, Giulia Di Stasio, Sabina Fontana

**Affiliations:** ^1^Department of Ancient and Modern Civilizations, University of Messina, Messina, Italy; ^2^Department of Humanities, University of Catania, Ragusa, Italy

**Keywords:** negation, Italian sign language, embodiment, Multiple Representation Theories, socio-semiotic approach

## Abstract

Negation can be considered a shared social action that develops since early infancy with very basic acts of refusals or rejection. Inspired by an approach to the embodiment of concepts known as Multiple Representation Theories (MRT, henceforth), the present paper explores negation as an embodied action that relies on both sensorimotor and linguistic/social information. Despite the different variants, MRT accounts share the basic ideas that both linguistic/social and sensorimotor information concur to the processes of concepts formation and representation and that the balance between these components depends on the kind of concept, the context, or the performed task. In the present research we will apply the MRT framework for exploring negation in Italian sign language (LIS). The nature of negation in LIS has been explored in continuity with the co-speech gesture where negative elements are encoded through differentiated prosodic and gestural strategies across languages. Data have been collected in naturalistic settings that may allow a much wider understanding of negation both in speech and in spoken language with a semi-structured interview. Five LIS participants with age range 30–80 were recruited and interviewed with the aim of understanding the continuity between gesture and sign in negation. Results highlight that negation utterances mirror the functions of rejection, non-existence and denial that have been described in language acquisition both in deaf and hearing children. These different steps of acquisition of negation show a different balance between sensorimotor, linguistic and social information in the construction of negative meaning that the MRT is able to enlighten.

## Theoretical Framework for the Analysis of Sign Languages

Our theoretical framework for the description of LIS is inspired by the socio-semiotic and cognitive model developed by Volterra et al. (2019) on Italian Sign Language (LIS) which, following the semiogenetic approach of Cuxac (2000), highlights the embodied and social basis of sign and spoken languages systems.

Humans communicate in a great variety of ways depending on the languages in their repertoire, their communicative needs, the semiotic resources in the context: for example, hearing people can integrate their speech with pointing and representative gestures that look very similar to signs (see for example, the Italian gesture for coffee which is similar to the LIS sign^1^ and deaf people can use mouth actions as complements to the signed utterance (Boyes-Braem and Sutton, 2001; Fontana, 2008; Fontana and Roccaforte, 2015).

In sign languages, the hands, along with the entire body and with facial expressions, become components of a language. The body and the hands are involved in various daily tasks, such as showing, giving and pointing, or in a series of actions such as enumeration, handling, representing objects or characters or actions performed by the characters (Boyes Braem, 1981). Different sign languages choose different representational strategies referring to the same object (see for example the sign for “to eat” in different sign languages; see text footnote 1). Beyond the lexical units (the so-called “frozen” or “standard” lexicon), complex referential expressions with highly iconic and simultaneous features have been identified. These units have been defined as “Highly Iconic Structures” (HIS), and further specified as Transfer of Person, Situation and Form (Cuxac and Antinoro Pizzuto, 2010). Sign languages express meaning in two different ways: (a) a depictive intention, that is “show, illustrate and demonstrate” by using HIS, and (b) a non-depictive intention, consisting of “telling” (without showing) by using the standard lexicon and pointing signs. In other words, sign languages are rooted in a process of iconization of signers’ perceptual-practical experience. These two different semiotic intentions are conveyed by the direction of the eye gaze: with standard signs, the eye gaze is directed toward the interlocutor, or toward certain points in space; in the case of the iconic structures, the gaze is directed toward the hands or it represents the gaze of the entities symbolized. Such strategies underlie both the production of signs and of gestures. Crosslinguistic research on the development of language (Marentette et al., 2016) have described four strategies of symbolic representation that have been later classified (Volterra et al., 2019) both in hearing and deaf children and adults which consists namely of: Own-body or enactment where the whole body represents the action or the character (imitating a cow, for example); Hand as hand with the hands assuming a grasping configuration that mirrors the action performed on the object shown (driving a car); Hand as object when the hands become the object (for example a ball); shape and size when the hands trace the shape or indicate the size of the object to be represented (draw a circle to represent a tower).

These strategies have been systematized in sign languages and are mirrored in the three mechanisms of signification recently described for LIS that are pointing, describing and depicting (Volterra et al., 2019) depending on the fact that a physical or social entity is in the context or not. Such embodied mechanisms can be noticed starting from the sub-lexical units which are: at the manual level, handshape, orientation, location movement; at the body level, mouth actions, facial expression, movement of the torso and gaze direction. One important effect of the role of human sensory motor experience in sign languages is iconicity. Types of iconic mapping may range from a form reproducing under a certain respect the referent to a form of iconicity requiring more abstract mapping of features (Perniss and Vigliocco, 2014).

At the same time, these strategies confirm embodied and grounded views according to which acting and interacting with physical and social entities and objects in the environment is the base of our cognitive abilities (Gallese and Lakoff, 2005; Barsalou, 2018; Cuccio and Caruana, 2019). There is a continuity between action, gesture and sign or word that reflects the various aspects of the cognitive structure underlying them. It has been suggested that the activation of motor neurons when we are not actively carrying out any motor act, has a constitutive role in the comprehension of language. Both the mechanism of simulation and the production of gestures while we are speaking can be considered expressions of the embodied nature of meaning (Marghetis and Bergen, 2014) and are tightly interconnected (Cuccio and Fontana, 2017). To date, a huge amount of experimental studies, carried out with several experimental techniques, have supported this embodied approach (Jirak et al., 2010; Cuccio and Gallese, 2018). In this framework, negation might also be grounded in the sensorimotor system and might recruit the neural mechanisms underlying motor response inhibition (e.g., Beltrán et al., 2018). Findings in support of this hypothesis have been provided both in behavioral and electrophysiological studies (for a discussion, Montalti et al., 2021). The latter (e.g., Beltrán et al., 2018) suggested that the processing of negation might modulate the activity of the right inferior frontal gyrus, an area known to play a role in inhibitory control (Aron et al., 2014). Many questions remain open in this debate. First, there is no sufficient evidence to conclude that the mechanism for motor inhibition is recruited during the processing of negation regardless of the sentence contents (i.e., we do not know whether the involvement of motor inhibition resources is specifically linked to the processing of action-related sentences or it also underpin the comprehension of abstract sentences) nor we know whether the motor inhibition mechanism is recruited regardless of the language modality (spoken or signed). We will explore negation in LIS since for their semiotic nature, sign languages can enlighten the continuity between gestures and sign and the role of multimodality and embodiment in expressing negation.

## Expressing Negation in Italian Sign Language

Generally, negation is described as a logico-linguistic device that enables us to deny what we speak about (Virno, 2013). However, negation is not only refusing, simulating or dissimulating something: it is above all *acting* a negation in terms of the functioning of the sensorimotor system. There is not only one way but several forms of negation. Various studies have shown that in sign languages negation occurs both at the manual and non-manual level. At the manual level, signed units whose position can differ from one sign language to another are used. At the non-manual level, headshakes and some kind of mouth actions can co-occur either with the lexical unit or with the entire utterance. Furthermore, specific lexical units with negative functions, often accompanied by mouth actions, have been described in various sign languages (see Hendriks, 2008; Pfau, 2015; Oomen et al., 2018 for a review). This does not mean that negation functions in similar ways across sign languages. Studies have shown that there are differences on the form and use of the manual and non-manual markers. According to Pfau (2008), non-manual negation markers consist only of side-to-side headshake. It can also consist of non-manual markers that originate from hearing gestures. For example, in the Eastern Mediterranean area (e.g., Greece and Turkey) and in the Middle East (e.g., Jordan), for negation the hearing population use a single backward movement of the head. Such a form of negation has been found also in Sicily, where it is used alongside the negative headshake. In LIS, negation has been described as a formal operator within the Generative Grammar framework (Geraci, 2006). Geraci (2006) describes various manual negative signs such as NO that usually comes at the end of the utterance. In his view, a clause cannot be negated by means of the headshake only, but should be always accompanied by the manual form.

The research conducted by Gianfreda (2010) was devoted to analyzing the linguistic expression of certainty and uncertainty in LIS. The corpus was based on conversations in LIS between deaf people communicating through a video-chat software in an informal context. Gianfreda has described some lexical forms of negation which in his view have gone through a process of grammaticalization such as the various forms of IMPOSSIBLE (pictures taken from Borghi et al., 2014). The sign glossed as IMPOSSIBLE-PA-PA refers to a condition of unfavorable circumstances for an action or an event due to some external event (an authority, for example). This negative sign co-occurs with a specific mouth gesture that is “a-pa.”


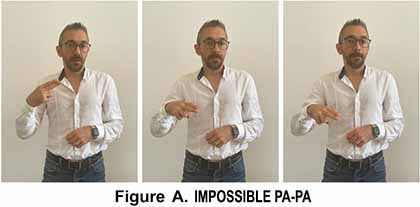


It seems to be derived from the sign FORBID which shares the parameters of the handshape and downward movement. However, in IMPOSSIBLE-PA-PA, the movement is repeated and more rapid. In the sign glossed as IMPOSSIBLE-fff based on extended fingers that move upward in a circular movement, any possibility for an event to take place are excluded. This sign is accompanied by the mouth gesture which corresponds to “air emission” and has been glossed as “fff.”


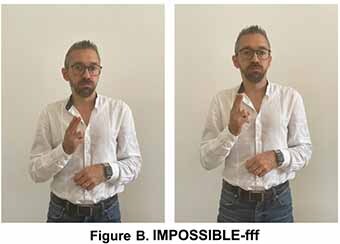


This sign seems to derive from the blessing gesture typical of Christian religion and is similar to a gesture of Southern Italy used for referring to a dead or dying person, also in metaphorical terms. It is worth noticing that this last variant has been incorporated into LIS as an autonomous lexical unit, i.e., the sign DEAD, produced without the mouth gesture “fff” which is co-produced in IMPOSSIBILE-fff. The LIS signs IMPOSSIBLE/POSSIBLE respectively convey the notion of absence of certain conditions or characteristics or the existence of actual or potential conditions for an action or event to take place.


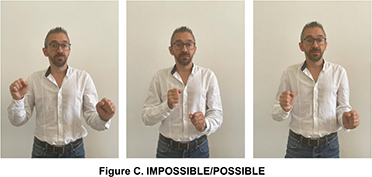


The two signs share the same hand configuration (two fists) but are executed with different movements (Wilcox et al., 2010; Gianfreda et al., 2014).

Such research show the various forms of negation in LIS: first, the sign NO/NON-that seems to function as a logical operator for denial; second, body components (facial expressions, head movements, mouth actions) that may act in co-occurrence with the manual signs; third, lexical units (e.g., the various forms of IMPOSSIBLE) that functions for negation and that go to head are strongly embodied in cultural and perceptual experience. The present study intends to explore negation as a form of action through MRT. We will show that negation is bodily grounded and multimodal and that it evolves out of the three steps in the acquisition of negation: (1) rejection/refusal; (2) disappearance/non-existence/unfulfilled expectation; (3) denial (Volterra, 1972; Beaupoil-Hourdel et al., 2016).

We hypothesize that negation involves the whole body together with manual signs and that the interaction between the sensorimotor, social and linguistic components can be fully understood if the various components of the negation action are analyzed as a global unit.

## Methodology

Data were collected through semi-formal interviews based on pictures to elicit the different negation actions in LIS.

The participants (see Table 1) are all part of the signing community and have been exposed to LIS since early childhood.

**TABLE 1:** Information about the participant.

Participants	Age	Gender	Profession
A	30–40	Male	LIS teacher
B	30–40	Female	Employee
C	80+	Female	Retired
D	40–50	Male	Employee
E	40–50	Female	Employee

Participants were invited to describe what they saw in the pictures. They were not asked specific questions on the pictures as we did not want to influence their answers. For this reason, drawing on everyday experience we proposed contrasting pictures that could elicit negation actions in a visual way. As shown below, pictures dealt with general topics such as health, health education, waste collection, healthy vs. unhealthy food, road speed limits and the safety of children.

Data have been glossed on a four-layer line that allow the representation of body components in order to highlight the co-occurrence of the various body and manual components.

sx________________________________________________

two handed________________________________________

dx_______________________________________________

body_____________________________________________

In this paper, we annotated LIS by using pictures that represented the entire utterances as shown in the examples below.

## Data Analysis

The analysis of data shows that the selected pictures do not always elicit negative units. In some cases participants choose to describe rather than to oppose the two contrasting pictures and use some form of negation. This is probably related to the fact that the participants were simply invited to describe the pictures in order not to influence them. This preliminary result proves to be interesting for further investigation at the level of the eliciting materials. For example, only two out of five participants systematically used negative units: participants A and B.

**FIGURE 1:**
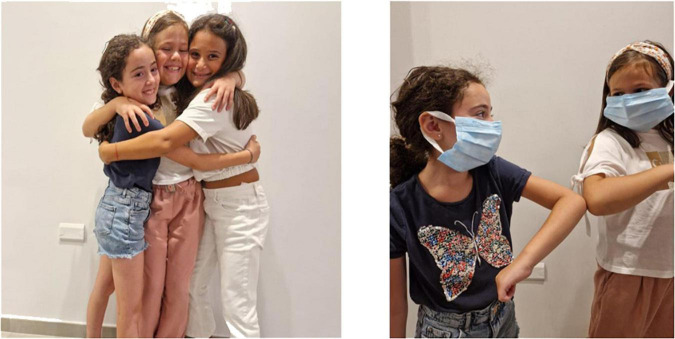
COVID-19 greeting.

The negative element of the utterance for Figure 1 THERE-IS-NOT co-occurs always with a specific facial expression and a mouth action. Depending on the discourse, the mouth action can consist of the mouthing “there is not” fully or partially articulated, or of the mouth gesture based on lips protrusion.

**Utterance 1:**
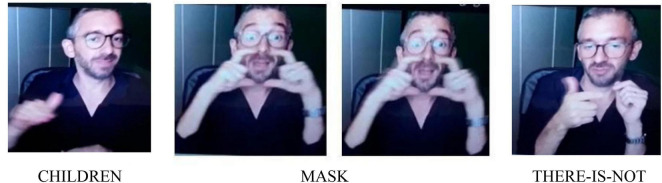
COVID-19 greeting.

The utterances of two participants (A and B) are based on a similar structure, although the two-handed sign THERE-IS-NOT, is articulated with one rather than two hands by one of the two participants, as shown above.

**FIGURE 2:**
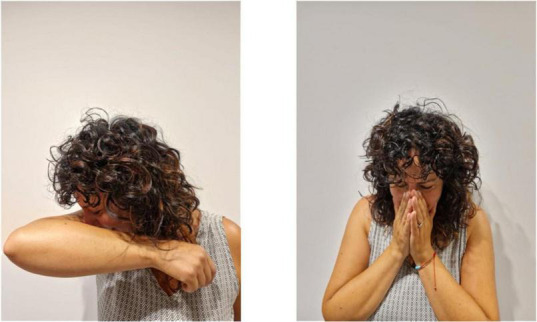
Sneeze.

The verb “sneeze” of the utterance 1 for Figure 2 is a transfer unit that reproduces the action of sneezing. The negation unit NO occurs at the end of the utterance and co-occurs with raised eyebrows and with the mouthing “no.”

**Utterance 2:**
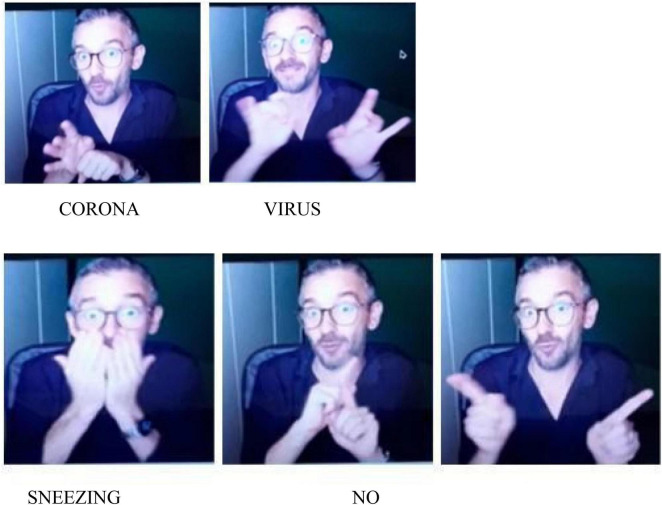
Sneezing before and after COVID-19.

Participant B does not use any negation unit in the utterance and simply explains that it is possible to sneeze outside. Another utterance for the Figure 2 is based on the following structure: SNEEZE—IN AIR—NO.

Other participants describe the pictures without using any element of negation.

**FIGURE 3:**
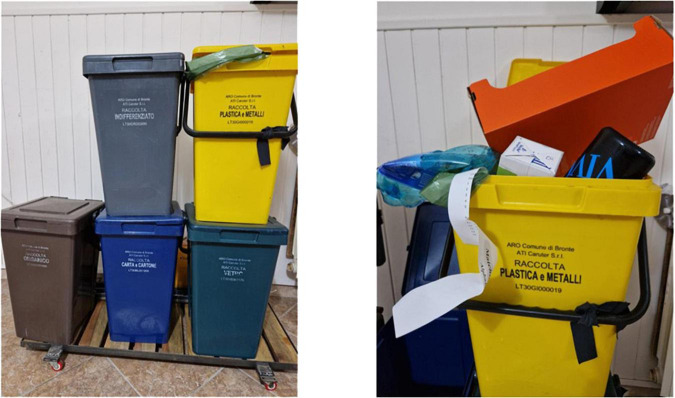
Separate waste collection.

Most participants simply described in Figure 3. Two participants use a particular facial expression with the signs BIN FULL to convey an avoidance effect.

**Utterance 3:**
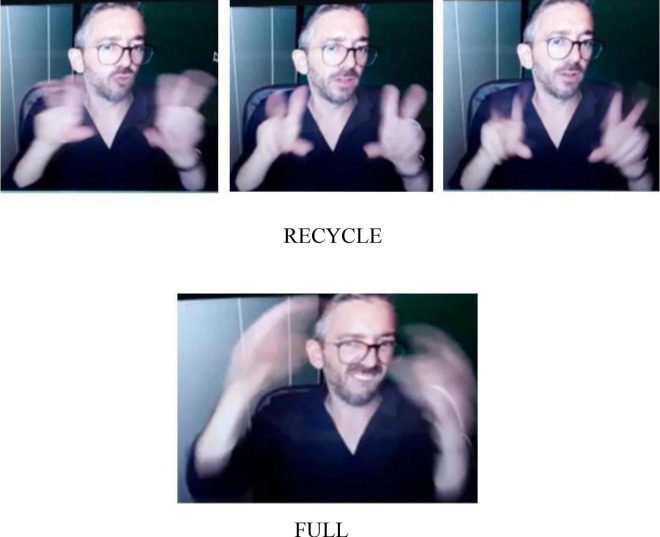
Separate waste collection.

**FIGURE 4:**
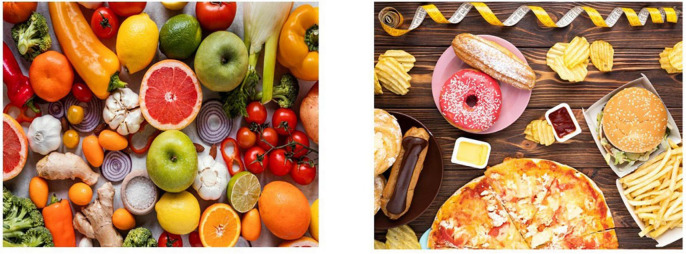
Healthy and unhealthy food. Available at: https://www.freepik.com/free-photo/assortment-healthy-unhealthy-food_5200655.htm.

In Figure 4, healthy and unhealthy food are opposed through the movement of the body on the left- and right-hand side respectively. The “junk food” information is followed by the negation unit NO.

**Utterance 4:**
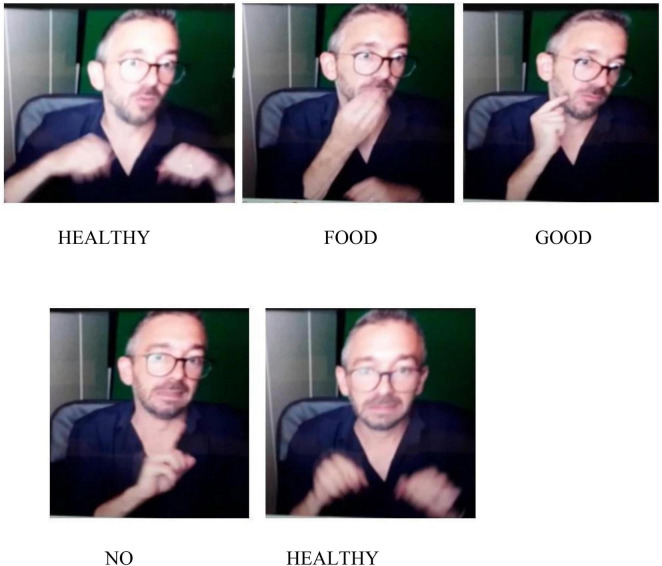
Healthy and unhealthy food.

An avoidance effect related to unhealthy food is conveyed by a shift in facial expression which is positive with the sign “healthy food” and negative with the sign “junk food” (Chen and Bargh, 1999).

The same utterance structure has been found in all participants. Participant C simply lists the food shown by the picture, using some personal examples in which no element of negation was found.

**FIGURE 5:**
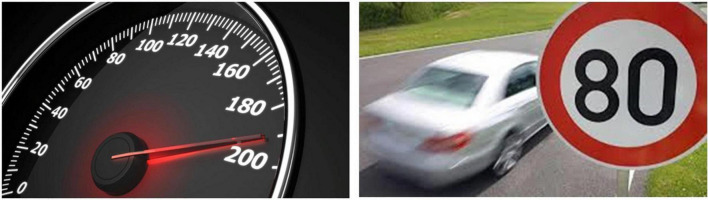
Road speed limit. Available at: https://www.pitstopadvisor.com/news/tachimetro-auto-effettiva-velocita/ and https://www.passiamo.lt/ll-llmite-dl-velocita-deve-ripetersl-dopo-ogni-intersezione-cass-civ-20-maggio-2014/.

Utterance 5 for Figure 5 is signed with a negation unit by only one participant. Other participants have chosen the lexical unit PROHIBITION or explained the reason why it was dangerous to drive fast. This picture seems to have elicited one more type of negation action that is related to a prohibition or to a specific request of not doing something^2^.

**Utterance 5:**
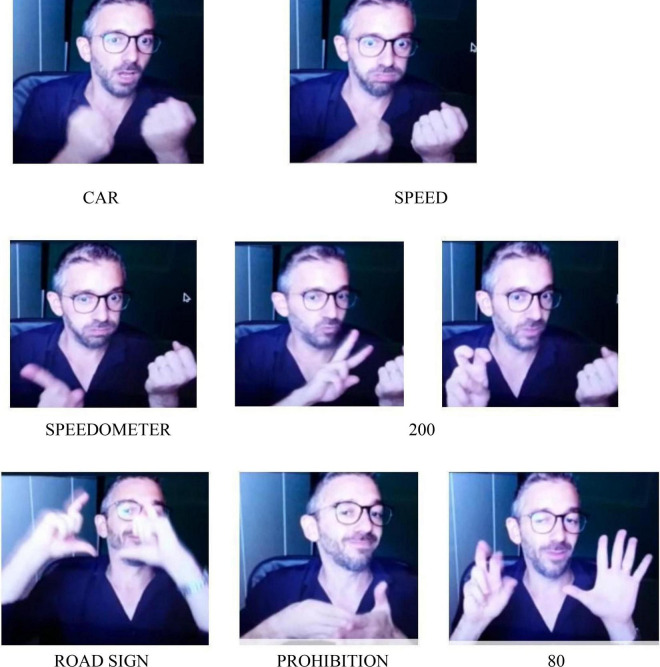
Road speed limit.

This utterance has been produced with an action of refusal/rejection that is conveyed by a backward movement of the body and a facial expression of “rejection.”

In another case the expression of rejection conveyed by a backward movement of the body co-occur with the entire utterance.

## Results

We have identified three different strategies to express negation in LIS that confirm that negation is bodily grounded and multimodal. In our opinion, negation actions in adults’ signing seem to mirror the three steps in the development of negation, previously mentioned: (1) rejection/refusal; (2) disappearance/non-existence/unfulfilled expectation; (3) denial (Volterra, 1972; Beaupoil-Hourdel et al., 2016).

**FIGURE 6:**
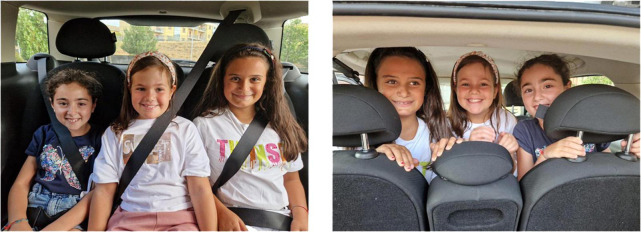
The safety of children in the car.

Such strategies can be classified in two formal categories following their form: logical-indexical or lexical. The various forms of negation always occur with different body components such as facial expression, mouth actions, torso backward movements. We consider them as forms of action with various degrees of embodiment. The logical category includes actions of denial which act at a linguistic level and refer to part or the whole utterance such as NO. They occur always at the end of the utterance or of the part of the utterance that has to be negated. Denials are negation actions that have a logic-pragmatic function as they act in the utterance with a linguistic aim. We maintain that the use of negation can negate the utterance content and have also metalinguistic and pragmatic functions related to the meaning. Lexical negation actions include negation units which play a stable lexical role within the utterance such as DO-NOT-LIKE, THERE-IS-NOT, NOT-YET. These negation units always occur with body components. As we have seen, either mouth gestures or mouthings can be used following the discourse needs. Both THERE-IS-NOT and NOT-YET can be considered as example of disappearance/non-existence/unfulfilled expectation and they seem to confirm the continuity between gestures and signs both in early infancy and in adult signing (Marentette, 2016; Volterra et al., 2019). In addition to this, prohibition is conveyed by specific lexical signs as in the utterances related to Figure 5 and can be somehow related to rejection. Nevertheless, they pragmatically imply different perspectives and they have a different cognitive and symbolic load. Indeed, rejection can be found in preverbal communication and also in animal communication whereas prohibition requires abstract mental representation (Cuccio, 2011). Finally, we have found an item of negation action exclusively on the body with the rejection/refusal action that co-occur with the torso backward movement together with the lexical unit “junk food” or “not wearing seat belts,” as in the utterances related to Figure 4 and Figure 6 although Geraci (2006) maintained that any form of non manual negation is always accompanied by a manual sign. These examples further enlighten the concept of avoidance posited by Chen and Bargh (1999).

**Utterance 6:**
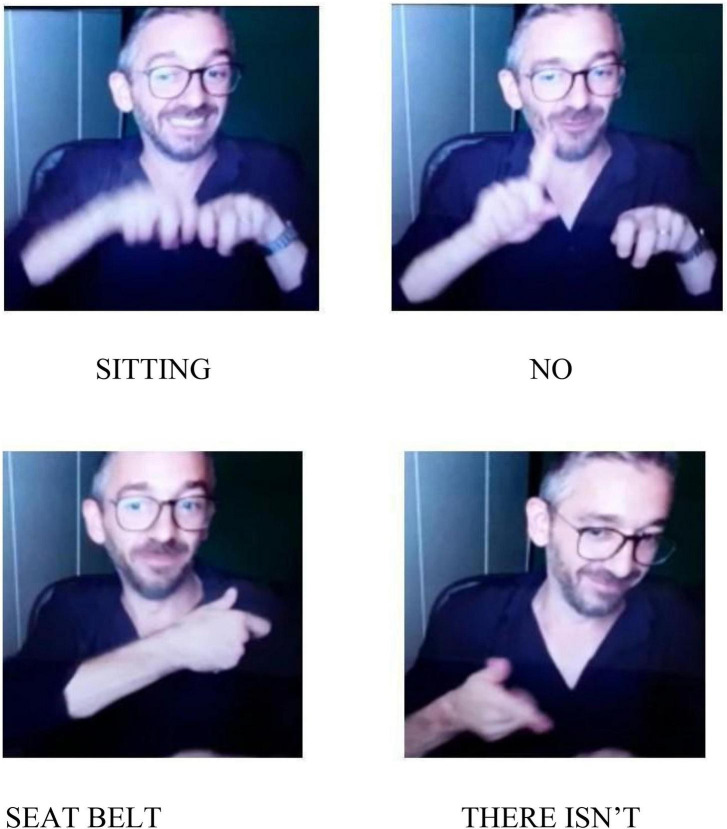
Child safety in the car.

Results of the present study prove that the use of pictures can be productive as it does not influence the signing structure and elicit the various forms of negation in LIS, but at the same time, it might not be effective when the participant chooses to describe pictures rather than using negation strategies. Even so, the data have shown that negation in LIS is far more complex than it has been described so far and that it is strongly linked on the one hand to the sensori-motor system and on the other to the logical structure of the utterance.

## Sensorimotor and Linguistic Components in the Expression of Negation: The Multiple Representations Account

As we have seen, in LIS, negation has been described mainly as a formal operator, or from a functional perspective within the utterance, although it involves various body components in continuity with motor action and gesture. Negation can be considered also a shared social action that develops since early infancy with very basic acts of refusals or rejection. The data discussed in the previous sections, although preliminary, confirm this perspective. The representation of negation in LIS and, generally speaking, the representation of negation in signed languages, seem to combine different kinds of information, ranging from bodily, multimodal and social information to purely linguistic information. Indeed, negation in LIS can be expressed through logical-indexical and lexical structures which co-occur with body components such as facial expressions, mouth actions and torso backward movements and can play the functions of rejection/refusal, disappearance/non-existence/unfulfilled expectation, denial. The lexical structures can be considered as forms of action with various degrees of embodiment. Our data, thus, confirm the idea that in LIS, as in all other signed languages, language is embodied not only inside (see below for a discussion of the role of the sensorimotor system in signed language comprehension) but also outside through the involvement of non-purely linguistic components in the process of meaning construction.

Hence, the recruitment of bodily components in signed languages’ negation is in line with an embodied account of language, which posits that the production/comprehension of language is grounded in our sensory-motor system (Di Cesare et al., 2017; Cuccio and Gallese, 2018; Gallese and Cuccio, 2018) and further extends this account to logical operators such as negation.

A vast amount of experimental data, in the last years, corroborated the hypothesis that systems for action and perception may also play a crucial role in the processing of different types of linguistic information (Fischer and Zwaan, 2008; Barsalou, 2010; Jirak et al., 2010; Mirabella et al., 2012; Glenberg et al., 2013; Cuccio et al., 2014; Pulvermüller et al., 2014; Spadacenta et al., 2014; Cuccio, 2022). This means that the processing of action and perception related linguistic expressions recruits the sensorimotor system. For example, the comprehension of the sentence “Mary grasps the glass” will activate hand-related areas in the premotor cortex. This mechanism is known as Embodied Simulation (Sinigaglia and Gallese, 2011). Sensorimotor information made available by the mechanism of simulation, in this view, will contribute to the construction of linguistic meaning. This embodied account of language refuses the classical, first generation, cognitive science view based on the idea that concepts and meanings are represented using amodal and abstract symbols (e.g., Fodor, 1983).

Recently, evidence for embodied processing in signed language users have also been provided. For example, in an electroencephalographic (EEG) study, Kubicek and Quandt (2019) showed that the sensorimotor system is recruited during signs processing. In this study, the authors assessed whether systems for action and perception are differently modulated by the observation of signs produced with, respectively, one and two hands. Results showed greater alpha and beta event-related desynchronization during the perception of two-hand signs compared to one-hand signs. Alpha and beta event-related desynchronization is likely due to motor simulation and is thus a mark of an embodied processing of signs. Thus, summarizing, findings from Kubicek and Quandt (2019) study showed that signs comprehension draws on sensorimotor information, too, determining the activation of the motor cortex. The latter was more extensively recruited by the processing of two-hand signs compared to one-hand signs. Sensorimotor information, in this case, too, contributes to the process of meaning construction. Thus, data seem to support the embodied view of language, independently of the language modality.

In this framework, recent findings have shown that also linguistic negation, which is thought of as an abstract and purely logico-linguistic operator, is grounded in the sensorimotor system. Indeed, it has been shown that the processing of negation recruits the mechanisms for motor response inhibition (e.g., Montalti et al., 2021). Behavioral (e.g., Montalti et al., 2021) and EEG (e.g., de Vega et al., 2016; Beltrán et al., 2018) studies supported the hypothesis of the embodiment of negation in motor inhibitory mechanisms. For example, de Vega et al. (2016) carried out an EEG study in which participants were asked to read negative and affirmative action-related sentences while performing a Go/NoGo task. The Go/no GO task is specifically designed to evaluate the recruitment of resources for motor inhibition and consists of a go-task and a noGo-task. Trials of the two tasks are randomly intermixed, the Go-trials are the most frequent type of trials and require the subject to respond (i.e., pressing a key on the keyboard) as fast as possible when a go-signal is presented. The noGo trials are less frequent and require the subject to withhold a response (not pressing any key). Findings from de Vega et al. (2016) study showed that negative sentences modulate theta bands, a marker of motor inhibition, over the frontal cortex. These data suggest that, to explain the processes underlying the construction of meaning in the expression of linguistic negation, very likely, we need to account for how different kinds of information, including sensorimotor knowledge, contribute to this process.

Although studies on the embodiment of negation in signed languages have not yet been conducted, in the light of the data discussed in the previous sections, showing that the expression of negation in LIS involves bodily, multimodal and social information together with purely linguistic information, we might hypothesize that the expression of negation is embodied independently of the language modality and that negation in sign languages might recruit, too, the mechanism for motor response inhibition.

It follows that we need to develop a model of how purely linguistic, social and sensorimotor information all contribute to and are balanced in the process of meaning construction, especially in the expression of negation, and we need to develop a model which can account for this process independently of the language modality.

We must admit that this is not an easy endeavor. To date, to develop an account of how sensorimotor information might interface with purely symbolic or social knowledge in the formation of abstract meanings, such as negation, is the most difficult challenge for any embodied approach to language. Lately, the issue of the integration of different kinds of information (i.e., sensorimotor, purely linguistic and social) in the representation of both concrete and abstract meanings/concepts has been addressed in the framework of the so-called *Multiple Representation Theories* (henceforth, MRT; e.g., Borghi et al., 2017). Different MRT variants are currently discussed. They differ in some respects, but all share the basic idea that concrete and abstract meanings/concepts representation relies on both sensorimotor, as well as on linguistic and social knowledge. The degree of involvement of these different sources of information has been differently accounted for and varies in relation to the kind of meaning/concept, the context, or the performed task.

In this paper, we framed our proposal following the Word as Social Tools (WAT) theory which is the account developed by Borghi et al. (2019) within the MRT approach. According to the WAT theory, abstract meanings/concepts are linguistically and socially acquired whereas the acquisition of concrete meanings/concepts rely mostly on perceptual similarity. Social and linguistic information is, thus, by large more important in the acquisition of abstract meanings/concepts. Since abstract and concrete meanings/concepts follow different trajectories of acquisition, they are also differently represented in the brain. Indeed, whereas both recruit the sensorimotor system, the networks underlying linguistic and social cognition are more activated by abstract concepts. More specifically, the WAT theory predicts that, since concepts/meanings are grounded in the same perceptual and motor systems that support their acquisition, abstract meanings/concepts processing determines the activation of the mouth (for spoken languages) and of the hands (for signed languages). This prediction has been confirmed by empirical data with regard to spoken languages (see Borghi et al., 2019). Data is still missing for signed languages.

Since the modality of acquisition impacts on the different representation of abstract and concrete concepts/meanings in the brain, it might be extremely useful to have a look at psycholinguistic data on the acquisition of negation. Psycholinguistics research has suggested that, independently of the language modality, there are at least three steps in the acquisition of negation: (1) rejection/refusal; (2) disappearance/non-existence/unfulfilled expectation; (3) denial (Volterra and Antinucci, 1979; Dimroth, 2010; Cuccio, 2011, 2012). Rejection is the first category of negation to be acquired and is used to express refusal of something in the present context. Examples of rejection can be found in human pre-linguistic gestures and even in animal behavior. Whereas rejection, according to Pea (1980) does not require abstract mental representation, non-existence and denial do require them. The second category of linguistic negation to arise is non-existence/unfulfilled expectation. At this point, children are able to signal the absence or disappearance of an expected referent in the context of speech or indicate something that violates their expectations, based on previous experience. Lastly, the third category to be acquired is denial which implies negation of a predication. The referent is usually symbolically expressed.

Following the MRT approach, we suggest that the acquisition of linguistic negation, in these three different steps, determines a path from concrete to more abstract meanings. Negation is initially acquired in the context of physical acts of refusal to later become an abstract and symbolic operator. Whereas rejection relies more heavily, although not exclusively, on sensorimotor information, linguistic and social knowledge is crucial especially for disappearance/non-existence/unfulfilled expectation and, most of all, denial. How these sources of knowledge are balanced depends on many factors, such as the context and the performed task.

## Conclusion

Summarizing, we know that the processing of negation recruits the motor inhibitory mechanisms and that, more generally, the comprehension of abstract meanings recruits the perceptual and motor systems which support their acquisition. Although we do have empirical findings on the embodied processing of signed languages, data are still missing on the embodiment of negation in this language modality. However, the data discussed in the previous sections suggest that the expression of negation in LIS exploits not only manual but also facial and bodily components whose role is in line with an embodied approach to language. In this light, this concluding section will be devoted to proposing an account of how different sources of information interface in the process of construction of meaning, especially abstract meanings such as negation. To this purpose, we will rely on Evans (2006) distinction between lexical concepts and meaning. In Evans’ view, lexical entries cannot be considered *per se* as the bearers of meaning. As Evans (2006) says (2006, 491), “[…] While lexical concepts constitute the semantic units conventionally associated with linguistic forms and form an integral part of a language user’s individual mental grammar, meaning is a property of situated usage-events, rather than words. That is, meaning is not a function of language *per se*, but arises from language use.” Linguistic meaning, thus, is much more than purely symbolic knowledge. It is always dependent on contextual factors and on the inferential processes underlying language production/comprehension (Carapezza and Cuccio, 2018). Lexical entries certainly contribute to the process of meaning construction, since symbolic knowledge is a crucial part of our ability to produce and comprehend language. Especially for abstract meanings, which are socially and linguistically acquired, they play a major role. In addition to this, lexical entries can be considered as cues that prompt us to activate our background, encyclopedic knowledge. Importantly, our encyclopedic knowledge includes different kinds of information: sensorimotor knowledge, social information, emotions and feelings. In this view, sensorimotor knowledge, being an integral part of our encyclopedic knowledge, constitutively contributes to the contextually based process of meaning construction. Evans (2006) account of linguistic meaning provides us with a framework to better understand how sensorimotor, social and purely linguistic information might interface. Within this perspective, we can easily envision that the balance between different sources of information is highly flexible and depends not only on the kind of concepts (e.g., concrete or abstract) but also on the context of use of that context and on the background knowledge of the speakers. In our proposal, this holds true also for the expression of negation.

Our results are preliminary. More research needs to be carried out to have a broader comprehension of the bodily grounding of negation in LIS and, generally speaking, in sign languages. Specifically, empirical studies on the embodied processing of this logical operator, with techniques such as the EEG, must be carried out. Furthermore, to have a better understanding of the differences and similarities between the communication of negation in deaf and hearing individuals, it would be extremely useful to compare how hearing participants would describe the very same stimuli used in the current study. These are the next points on our research agenda.

## Data Availability Statement

The original contributions presented in the study are included in the article/supplementary material, further inquiries can be directed to the corresponding author/s.

## Ethics Statement

Ethical review and approval was not required for the study on human participants in accordance with the local legislation and institutional requirements. Written informed consent for participation was not required for this study in accordance with the national legislation and the institutional requirements. Written informed consent was obtained from the individual(s) for the publication of any potentially identifiable images or data included in this article.

## Author Contributions

SF wrote the sections “Theoretical Framework for the Analysis of Sign Languages,” Expressing Negation in Italian Sign Language,” and “Results.” SF and GDS wrote the sections “Methodology” and “Data Analysis.” VC wrote the sections “Sensorimotor and Linguistic Components in the Expression of Negation: The Multiple Representations Account” and “Conclusion.” All authors discussed and designed the article together.

## Conflict of Interest

The authors declare that the research was conducted in the absence of any commercial or financial relationships that could be construed as a potential conflict of interest.

## Publisher’s Note

All claims expressed in this article are solely those of the authors and do not necessarily represent those of their affiliated organizations, or those of the publisher, the editors and the reviewers. Any product that may be evaluated in this article, or claim that may be made by its manufacturer, is not guaranteed or endorsed by the publisher.

## Footnotes

1
https://www.spreadthesign.com/it.it/search/

2 We thank reviewer one for this suggestion.
